# Ethical Considerations When Designing and Implementing Immersive Realities in Nursing Education

**DOI:** 10.7759/cureus.64333

**Published:** 2024-07-11

**Authors:** Elizabeth V Sombilon, Shai S Rahmanov, Kayla Jachecki, Zipora Rahmanov, Eva Peisachovich

**Affiliations:** 1 Nursing, York University, Toronto, CAN; 2 Environmental Studies, York University, Toronto, CAN; 3 Health, York University, Toronto, CAN; 4 Medical Education and Simulation, York University, Toronto, CAN

**Keywords:** ethics and professionalism, clinical empathy, ethical and equity consideration, nursing education and practice, immersive technologies

## Abstract

Immersive realities such as Augmented Reality (AR), Virtual Reality (VR), Mixed Reality (MR), and all future realities yet to be introduced, offer transformative potential as a new teaching methodology. VR and AR technologies in nursing education provide immersive experiences to students and allow them to practice clinical skills in a safe and controlled learning environment. However, VR and AR developers, along with educators who create simulated scenarios, must address ethical considerations such as accessibility, diversity, and inclusion in the design and implementation processes. While these innovative technologies are promising, they must be offered in a safe and effective manner. This editorial delves into ethical considerations that are involved in designing and implementing AR and VR as immersive realities in nursing education.

## Editorial

In the realm of education, immersive realities can be used as an umbrella term for Augmented Reality (AR), Virtual Reality (VR), Mixed Reality (MR), and all future realities yet to be introduced that offer the potential of transformative non-traditional teaching methodologies. Extended reality is used to describe the full spectrum of real and virtual environments generated by computer technology and wearables. These technologies enable users to enhance their critical thinking and problem-solving skills while working among educators and colleagues and have the potential to augment learning and application from theory to practice.

Immersive realities are computer-based technologies that are used to enhance the interactive experience and integrate real and virtual environments, creating immersive digital experiences that replace the real world, whilst allowing users to interact with both digital and real elements. Extended realities serve as a medium to support experiential education and offer the ability to transfer lessons into clinical settings without setting foot in a healthcare institution. 

VR is a technology that creates a fully immersive, simulated digital environment that replaces the user's real-world surroundings. It is experienced through a headset that blocks out the physical world and overlays visuals onto the real world. VR can be used for various applications such as gaming, training simulations, virtual tourism, and social interactions, aiming to create a convincing and interactive experience that makes the user feel as though they are truly "inside" the virtual world. For instance, nursing students can use VR headsets to practice clinical skills such as drug administration, catheter insertions, and phlebotomy procedures, allowing for safe and effective hands-on learning.

AR, on the other hand, overlays digital information and virtual objects onto the real-world environment, enhancing the user's perception by superimposing computer-generated sensory inputs such as visuals, sounds, or global positioning system (GPS) data onto their view of reality. AR is typically experienced through devices like smartphones, tablets, or AR glasses and finds applications in mobile apps, educational tools, navigation systems, and retail experiences. Unlike VR, which substitutes the real world with a fully synthetic environment, AR enriches the existing real-world environment with virtual elements. For instance, AR headsets can overlay additional visuals onto real-world models to augment the visualization of organs relevant to cardiopulmonary assessments. This technology enables students to enhance their physical assessment skills by providing guidance in locating landmarks when assessing the heart, lungs, and thorax. 

MR combines elements of both VR and AR, allowing real and virtual elements to interact in real time. In MR, users can interact with and manipulate both physical and virtual items and environments using next-generation sensing and imaging technologies. For instance, a user might place a virtual object on a real table and interact with it as if it were a tangible object, blending the boundaries between physical and digital realms.

Immersive realities are transforming various industries, including entertainment, education, healthcare, and manufacturing, by providing new ways to visualize, simulate, and interact with digital information and environments. The advancement of immersive realities is continually pushing the boundaries of how we perceive and engage with the world around us, making it a pivotal technology for the future.

Theoretical frameworks 

Immersive realities have increasingly been integrated into nursing education to enhance the learning experience through simulation-based training. The immersive experiences provided by these virtual, augmented, and mixed realities create a safe and controlled environment for students to refine their clinical skills [1] and provide realistic patient scenarios that replicate physiological and emotional responses for learners. VR can simulate a wide range of patient conditions with which students can interact in order to refine their clinical decision-making skills. Sharing thoughts and ideas with peers and instructors during time-outs and debriefs can further guide students during the scenario and prepare them for their clinical practice. Students can also engage in preliminary learning by observing their peers while they wait for their turns. This vicarious learning occurs as peers evaluate each other’s performances; the neural processing that occurs during vicarious learning runs a similar pathway to that of learning from trial and error [2]. This immersive state also provides users with a sense of spatial presence, which encourages and cultivates a space for students to willingly participate in active learning. Empathy, communication, adaptability, and collaboration are examples of skills that can be molded using simulations [1]. Immersive realities invite students to learn from their mistakes without causing harm to real patients, as scenarios can be repeated to incorporate immediate feedback from instructors. While these immersive realities offer promising learning opportunities, educators must consider ethics when designing and implementing scenarios.

Ethical considerations and nursing education 

Immersive realities have the potential to enrich morality in nursing education, as these innovative tools offer immersive experiences that nurture and cultivate ethical, compassionate, and empathetic decision-making skills. Immersive realities have vast potential to transform nursing education by providing students with realistic and engaging learning experiences. VR simulations can recreate realistic patient-care scenarios, allowing nursing students to practice clinical skills in a safe and controlled environment by interacting with virtual patients, assessing their condition, and making decisions about their care, all while receiving feedback and guidance from instructors.

AR can revolutionize nursing education by overlaying digital information onto actual body structures. For instance, holding a tablet over a body part can superimpose digital information about specific structures, identifying their functions accordingly, and other relevant details. Nursing students benefit significantly from AR applications that allow them to visualize and interact with 3D models of the human body. This technology provides them with opportunities to grasp complex anatomical structures and understand their relationships in a more tangible and interactive way. Moreover, AR blends elements of both VR and AR itself, enabling nursing students to practice essential skills. They can interact with virtual patients overlaid onto the real world, facilitating practice in communication and interpersonal skills crucial for patient care, such as empathy, active listening, and cultural competency.

Through VR and AR simulations, nursing students can experience the perspectives of patients in specific scenarios, helping them gain a deeper understanding of patients’ perceived realities. Providing students with an opportunity to navigate through complex ethical dilemmas while receiving guidance in a safe environment can instill the values that surround patient-centered care. Immersive realities can facilitate interprofessional education by allowing nursing students to collaborate with students from other interdisciplinary fields in simulated patient care scenarios. This helps foster teamwork, communication, and mutual respect among future healthcare professionals. 

When integrating immersive realities into nursing education, several ethical considerations must be addressed to ensure the well-being of students, patients, and other stakeholders. Key ethical considerations include informed consent, privacy and confidentiality, avoiding harm, cultural sensitivity, accuracy and quality of content, equitable access, and professional boundaries.

The use of VR and AR in immersive learning experiences represents a departure from traditional real-world simulations where actors serve as patients. While simulations with actors offer valuable training opportunities, VR and AR technologies provide distinct advantages. They enable nursing students to engage with virtual patients and anatomical models in a controlled, repeatable environment that enhances learning and skill development. For instance, AR can overlay detailed anatomical information directly onto anatomical and clinical specimens, facilitating a deeper understanding of complex structures.

In these immersive experiences, it is paramount that students give informed consent before participation, particularly in scenarios involving VR simulations or AR interactions that may elicit strong emotions or discomfort. Unlike simulations with actors, immersive technologies can immerse students deeply in realistic scenarios, potentially triggering emotional responses. Informed consent ensures that students are aware of the nature of the experience, its potential impacts, and their right to withdraw if necessary. This ethical practice fosters a supportive and respectful learning environment where students can engage effectively and responsibly with emerging technologies. It is important that students provide informed consent before participating in immersive learning experiences, especially those involving VR simulations or AR interactions that may evoke strong emotions or trigger discomfort. Clear information about the purpose, risks, and benefits of using immersive realities should be provided, and students should have the option to opt out if they feel uncomfortable.

Privacy and confidentiality must also be considered as immersive realities may involve the use of personal data, such as biometric information or performance metrics collected during simulations. It’s essential to protect this data, ensuring that it is securely stored, anonymized when possible, and used only for educational purposes.

Immersive learning experiences should be designed to minimize the risk of physical or psychological harm to students and patients. These responses include cybersickness, with symptoms including nausea, vomiting, eyestrain, disorientation, ataxia, vertigo, an altered sense of reality, and general discomfort. Simulations should be realistic but not overly distressing, and appropriate support should be available for students who may experience emotional distress or discomfort. VR and AR can sometimes be more realistic than expected, as immersive realities can access the user’s body and actions and thus breach the user’s physical privacy. VR and AR experiences should be culturally sensitive and inclusive and consider the diverse backgrounds and perspectives of nursing students and patients. Care should be taken to avoid stereotypes, biases, or offensive content that could harm cultural or social groups. VR and AR simulations used for nursing education should be based on evidence-based practice and accurate medical information.

To ensure the accuracy and quality of content, it should be reviewed by subject-matter experts to ensure educational quality and clinical relevance, thus minimizing the risk of misinformation or incorrect training. Efforts should be made to ensure equitable access to immersive learning technologies for all students, regardless of their socioeconomic status, disabilities, or geographic location. This may involve providing financial support for hardware and software, offering alternative learning modalities for students with disabilities, or ensuring that simulations are accessible to students in remote or underserved areas. Immersive simulations should also reinforce professional boundaries and ethical standards in nursing practice. Students should understand the importance of maintaining patient confidentiality, respecting cultural and religious beliefs, and upholding ethical principles such as beneficence, nonmaleficence, and justice. By addressing these ethical considerations, nursing educators can harness the potential of immersive realities to enhance learning experiences while upholding ethical standards and promoting the well-being of students and patients alike. 

Another ethical concern faced by educators in traditional practicum settings is ensuring that learners who have autonomy over their actions treat real patients in an ethical manner. While VR and AR tools address concerns regarding the ethical treatment of real patients, it is nonetheless important that educators embed consequences should students engage in immoral actions such as physical or psychological abuse to the simulated patient. Simulated actions or reactions without repercussions can lead to immoral deeds, defeating the purpose of enhancing the students’ ethical compass.

It is unclear who is legally responsible for the psychological symptoms experienced by VR users. Although there are ethical issues that surround the use of VR, there is minimal information about the perception of educators who collaborate with designers when creating their scenarios. Steele et al. [3] suggest that designers and educators consider point checks from initial conception to the final product. This means that the curriculum should undergo research to support its usability, with documentation of processes along the way. The practice of value levers and documentation can help identify personal biases and decrease the possibility of unintentionally introducing them into the curriculum. This practice indicates that theoretical frameworks can be used to guide the design and implementation of VR and AR in nursing education. 

Ethical responsibility for accessibility 

The ethical integration of AR and VR technology in education necessitates a profound commitment to accessibility for all users. However, there is a noticeable gap in the research and efforts to address accessibility barriers in this space. This gap highlights the pressing need to investigate and address these barriers to ensure that immersive educational experiences are accessible to everyone. 

Understanding diverse user needs is crucial for designing and implementing inclusive VR and AR environments. It begins with identifying potential barriers that could hinder users from fully experiencing or interacting with the design. Designers must become familiar with accessibility requirements, challenge assumptions about uniform control usage, and consider how to make environments accessible for users with disabilities such as visual, auditory, and motor impairments. For example, users with visual impairments could benefit from VR environments that integrate audio descriptions or tactile feedback to navigate virtual spaces effectively. AR applications can use audio cues or haptic feedback to enhance navigation for users with limited vision. Similarly, users with auditory impairments may require visual cues or subtitles incorporated into VR experiences to understand spoken instructions or cues.

Moreover, users with motor impairments might need VR controllers with customizable button layouts or adaptive input devices that accommodate different levels of dexterity. AR interfaces could incorporate gesture recognition or voice commands as alternative input methods, allowing users with limited mobility to interact more fluidly with virtual content. By designing with inclusivity in mind, AR and VR technologies can provide meaningful experiences for users across a spectrum of abilities, ensuring that everyone can participate and engage fully in immersive environments.

Diversity and inclusion 

Integrating diversity into VR and AR experiences is ethically critical in nursing education in order to prepare nurses adequately for real-world healthcare settings.

Peck et al. [4] have brought to light a significant disparity in participant representation in VR research, including the exclusion of minority groups. This gap diminishes the effectiveness and accessibility of VR applications: the underrepresentation of individuals with diverse identities in VR studies distorts our understanding of user experiences, thus hindering the creation of inclusive VR applications tailored to meet the needs of all users.

Additionally, Hayes and Johnson [5] emphasize the importance of using representative avatars in VR learning environments. They argue that including diverse representations of the embodied self through avatars enhances engagement and facilitates the transfer of behavior outside of virtual space [5]. This is supported by Peck et al. [4], who advocate for actively considering diverse perspectives in the design process as a means to break the non-inclusive cycle in VR research and create VR experiences that are inclusive and beneficial for everyone.

The underemphasis on diversity within the VR and AR space has led to a variety of undesirable user experiences. For instance, gender disparities are evident in spatial immersion, where female users often experience simulator sickness more frequently than male users. This disparity can result from differences in visual processing or vestibular sensitivity between genders, which are not always adequately accounted for in the design.

Furthermore, issues related to race, culture, and ethnicity are frequently overlooked in VR and AR studies. Research indicates that cultural backgrounds significantly influence virtual experiences [4]. For example, individuals from diverse cultural backgrounds may have varying expectations, preferences, or responses to virtual environments based on their cultural norms and experiences. These examples underscore how the lack of diversity considerations can lead to discomfort, disorientation, or disengagement among users who do not fit within the dominant demographic profiles typically studied or addressed in VR and AR development. Addressing these factors is essential for creating inclusive and engaging virtual experiences that resonate with a broader range of users.

When discussing diversity dimensions in VR and AR, it is critical to actively avoid reinforcing stereotypes, biases, or assumptions that may be offensive to diverse communities. Stereotypes and biases can manifest in various ways, such as gendered or racial assumptions about capabilities or preferences. For example, assuming that women are less technologically savvy than men or that individuals from certain cultural backgrounds have uniform preferences for virtual experiences.

Educators must aim to break the cycle of exclusion in VR research and design experiences that are inclusive by offering opportunities to challenge and correct these biases. For instance, immersive simulations can provide personalized learning experiences that cater to individual learning styles and preferences, regardless of gender or cultural background. By allowing users to explore diverse perspectives and environments in a safe and controlled setting, VR and AR can promote empathy, understanding, and appreciation for different lived experiences.

Theoretical frameworks

Several frameworks can be used in the design process for VR and AR in nursing education. Guiding frameworks for implementing immersive realities are codes of ethics, constructivism, case-based learning (CBL), and the clinical reasoning process. 

The first framework is the professional code of ethics, which presents recommendations for ethical behavior toward individuals. By consulting with outside ethicists, educators can design relevant scenarios in the earliest phases of the process [3]. Ethicists can improve the ethical quality of care practices by providing educators with theoretical insights and identifying elements of effective patient-centered care for practice-inspired scenarios. Ethicists and educators can discuss the philosophical and moral questions behind the type of scenario they aim to create, as well as how the simulation may affect users.

The second framework, constructivism, is defined as acquiring knowledge through assimilation. Social constructivism places a strong emphasis on social interactions in the learning process. This framework suggests that there is a connection between societal issues and technology in our lives. When immersive realities are incorporated into nursing education, considering what is socially acceptable to use is essential [3]. Once designers and educators have solidified an ethically sound and acceptable reality, they can apply CBL. 

The third framework is the case-based learning method that uses realistic events to present a problem and invoke problem-solving and interpersonal skills through active learning. With ethical considerations in mind, designers and educators can solidify the simulated scenario with CBL principles.

The fourth framework, the clinical reasoning process, involves the collection of information and identification of problems and issues to determine the scenario’s ability to meet established goals and outcomes. As students and learners immerse themselves in VR and AR simulations, educators can gather information pertaining to the system's usability and feasibility by considering users’ perceptions and gauging whether their critical thinking has improved.

Conclusion 

The integration of immersive realities into nursing education offers a promising method for improving critical reasoning, problem-solving, and interpersonal skills. It can also guide an individual’s praxis and enhance patient-care outcomes. While these technologies invoke active participation, designers and educators using them must think critically about how the simulated scenarios can affect users. Since it is unclear who is legally responsible if users suffer psychological or physiological experiences, it is up to designers and educators to prevent this from occurring. By following ethical guidelines that respect the dignity, rights, and physical privacy of users and foster social competence through the use of constructivism, immersive realities can be used to enhance nursing education. Ethical considerations should be an integral part of the design, implementation, and evaluation of system usability; by incorporating an interdisciplinary team of technologists, nurses, ethicists, and students in this process, immersive realities can be designed to support the future education of nurses. 
